# DNA Double-Strand Breaks Induced by Cavitational Mechanical Effects of Ultrasound in Cancer Cell Lines

**DOI:** 10.1371/journal.pone.0029012

**Published:** 2012-01-03

**Authors:** Yukihiro Furusawa, Yoshisada Fujiwara, Paul Campbell, Qing-Li Zhao, Ryohei Ogawa, Mariame Ali Hassan, Yoshiaki Tabuchi, Ichiro Takasaki, Akihisa Takahashi, Takashi Kondo

**Affiliations:** 1 Department of Radiological Sciences, University of Toyama, Toyama, Japan; 2 Carnegie Laboratory for Physics, Division of Molecular Medicine, University of Dundee, Dundee, Scotland; 3 Division of Molecular Genetics Research, Life Science Research Center, Graduate School of Medicine Pharmaceutical Sciences, University of Toyama, Toyama, Japan; 4 Advanced Scientific Research Leaders Development Unit, Gunma University, Gunma, Japan; University of Massachusetts Medical School, United States of America

## Abstract

Ultrasonic technologies pervade the medical field: as a long established imaging modality in clinical diagnostics; and, with the emergence of targeted high intensity focused ultrasound, as a means of thermally ablating tumours. In parallel, the potential of [non-thermal] intermediate intensity ultrasound as a minimally invasive therapy is also being rigorously assessed. Here, induction of apoptosis in cancer cells has been observed, although definitive identification of the underlying mechanism has thus far remained elusive. A likely candidate process has been suggested to involve sonochemical activity, where reactive oxygen species (ROS) mediate the generation of DNA single-strand breaks. Here however, we provide compelling new evidence that strongly supports a purely mechanical mechanism. Moreover, by a combination of specific assays (neutral comet tail and staining for γH2AX foci formation) we demonstrate for the first time that US exposure at even moderate intensities exhibits genotoxic potential, through its facility to generate DNA damage across multiple cancer lines. Notably, colocalization assays highlight that ionizing radiation and ultrasound have distinctly different signatures to their respective γH2AX foci formation patterns, likely reflecting the different stress distributions that initiated damage formation. Furthermore, parallel immuno-blotting suggests that DNA-PKcs have a preferential role in the repair of ultrasound-induced damage.

## Introduction

Ultrasound (US) is indispensable in most medical fields: (i) US at very low intensities (<0.1 MPa acoustic pressure) far below the thresholds for posing thermal and/or cavitational adverse effects is used for medical diagnosis; (ii) high intensity focused US (HIFU, >10 MPa acoustic pressure) is used for thermal ablation of tumors; and (iii) non-thermal low-intensity US (0.1–1.5 MPa acoustic pressure between the above two) as a potential candidate for cancer therapy is currently under research [1]. Tissues exposed to US energy can elicit a spectrum of biological response, each with distinct therapeutic potential [1]–[6], including uptake of exogenous molecules [7]–[14], necrosis, and apoptosis [1], [3], [6], [15], [16]. The biophysical modes of US are divided into three classes, thermal, cavitational, and non-thermal non-cavitational effects. Cavitation leads to a variety of mechanical stresses such as shear stress, shock wave, high pressure, and chemical stress due to free radicals formation, both of which have been inferred to act simultaneously on all biological materials [15]–[17]. Accumulating evidence indicates that intense US as well as low-intensity US excluding thermal effect induce reactive oxygen species (ROS) production, membrane fluidity, DNA single strand breaks (SSBs) and several previous studies implied the importance of SSBs arising from sonochemically produced ROS as DNA damage initiating US-induced cell killing/death [2]–[4], [6], [15]. However, this view is questionable, because numerous SSBs induced, for example, by the mmol/L range of H_2_O_2_ lead to no or very few double-strand breaks (DSBs), the most cytotoxic lesions of DNA [18]. To date, however, there is no direct evidence on DSBs induction and whether subsequent activation of DNA damage response (DDR) pathways might occur after exposure to US. In obvious contrast, data on the cellular response to ionizing radiation (IR), including induction of DSBs and downstream DNA damage response (DDR) have been more extensively reported [19]. Here, we address this point evaluating the genotoxic potential of low-intensity US. In our study, we assessed several definitive endpoints associated with the formation and processing of DNA damage, including DSBs, post exposure to US with a set of experiment carried out in parallel with IR irradiation sering as positive controls.

## Results and Discussion

**Figure 1:**
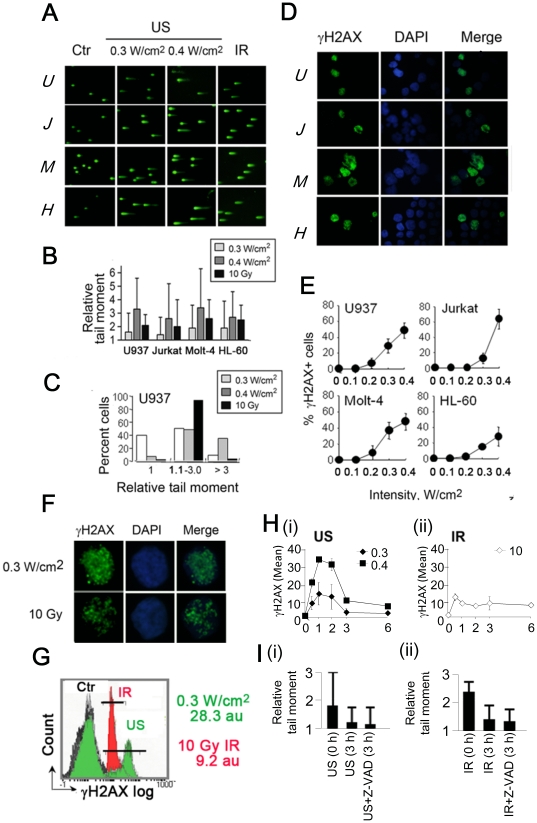
Induction and repair of DSBs and γH2AX foci after US or IR. (A) SYBR green-stained neutral comet tails immediately after exposure of U937 (*U*), Jurkat (*J*), Molt-4 (*M*) and HL-60 (*H*) cells to US (0.3 or 0.4 W/cm^2^) or IR (10 Gy). (B) Relative tail moments (*n* = 100 cells, means ± SD), normalized to the respective untreated controls ( = 1.0). (C) Heterogeneous distribution to >3.0, 1.1∼3.0 and 1 ( = control level) mean relative tail moments after US, but a uniform distribution to 1.1–3 relative tail moment after 10 Gy in ∼90% U937 cells (*n* = 100 cells). See Fig. S1 for other cell lines. (D) Green-fluorescentγH2AX images in U937, Jurkat, Molt-4 and HL-60 cells 30 min after 0.3 W/cm^2^ US (control cell images were not shown due to no γH2AX+ cells). (E) Induction of γH2AX+ cells as a function of US intensity beyond a threshold of 0.1–0.2 W/cm^2^ (*n* = 3, mean ± s.d.). (F) γH2AX+ cell images and (G) FCM histograms of γH2AX+ U937 cells 30 min after 0.3 W/cm^2^ and 10 Gy IR. Black, green, and red profiles are for control, US, and IR, with MFIs of γH2AX+ cells (5–100 γH2AX log)). (H) Induction/decline of γH2AX+ U937 cells (FCM) with time after 0.3, 0.4 W/cm^2^ (i) and 10 Gy IR (ii). (I) Reduction in tail moments during 3 h post- 0.3 W/cm^2^ US (i) or 10 Gy IR (ii). zVAD-fmk at 50 µmol/L was used to eliminate apoptotic DSBs.

Neutral comet tail assaying (NCTA) was utilized to detect DNA DSBs occurring in four different leukemia lines (U937, Molt-4, Jurkat, and HL-60), that had been subjected either to IR (10 Gy, unless specified otherwise), or US (as exposures using intensities of 0.3 or 0.4 W/cm^2^ lasting 1 minute). Positive results, in terms of extended comet tails compared with non-irradiated controls, were observed across all cell lines measured in the period immediately following exposure (t = 0) (Figure 1A). Quantitative comparison, in terms of the average relative comet tail moment (RCTM) arising (Figure 1B) produced the following trend across all cell lines: RCTM_0.4US_>RCTM_IR_>RCTM_0.3US_, which underscores the comparability of the respective US and IR doses chosen for this investigation, in terms of their facility to induce similar levels of DNA damage. Interestingly however, we noticed that IR produced average RCTMs predominantly within the range 1.1–3, whereas US exposures give rise to a wider range of resultant RCTM, the distribution for which was also a function of US intensity (Figure 1C and Figure S1).

Whereas NCTA analyses are regarded as DSBs, the presence of distinct γH2AX foci can also represent a definitive signature for DSBs [20]. We observed such γH2AX staining in all cells exposed to 10 Gy (Figure 1F), and importantly, in all cell lines exposed to US (Figure 1D) above a threshold intensity of circa 0.1–0.2 W/cm^2^ (Figure 1E and Figure S2, S3), indicating, for the first time, that US exposure might induce DSBs and thus present a tangible genotoxic risk.

Post-exposure observation on cells exposed to IR compared favourably with previous reports [21] in that γH2AX+ cells exhibited discrete foci distributed across the nucleus (Figure 1F), and also that subsequent temporal profiling of the γH2AX+ population exhibited peaking at 30 minutes post-exposure, followed by gradual decay (Figure S4). Notably, this latter reduction in total γH2AX+ populations tallied qualitatively with trends also observed using NCTA (Figures 1H(ii) and 1I(ii)), supporting the repair of IR-induced DSBs.

On US-exposed (insonated) cells, the relative fraction of γH2AX+ was more pronounced, as confirmed by flow cytometry, where γH2AX+ levels were approximately threefold higher compared to IR exposed cells (Figure 1G). Affected cells also exhibited a pan-nuclear γH2AX+ distribution, with occasional but distinct foci superimposed (Figure 1F). Interestingly, the γH2AX+ population peaked at 60 minutes post-exposure for both the 0.3 and 0.4 W/cm^2^ US cases employed, followed by a recovery period that plateaued after 6 h (Figure 1H(i) and Figure S4).

The obvious differences in typical γH2AX+ coverages arising for IR and US exposed cells, together with their distinctive relative comet tail moment distributions (Figure 1C), and significantly different γH2AX+ peaking times, suggests that their respective DDR signaling pathways are different in nature. Moreover, in cases where US exposure was applied, employment of the pan-caspase inhibitor z-VAD-fmk to suppress apoptosis appeared to have negligible effect (Figure 1I(i) and Figure S5), whereas TRAIL-induced γH2AX (caspase-mediated γH2AX) for example could be abolished (Figure S5): persuasive evidence that the observed induction and post-peak loss of γH2AX in all cases is likely associated with DNA damage and repair.

**Figure 2:**
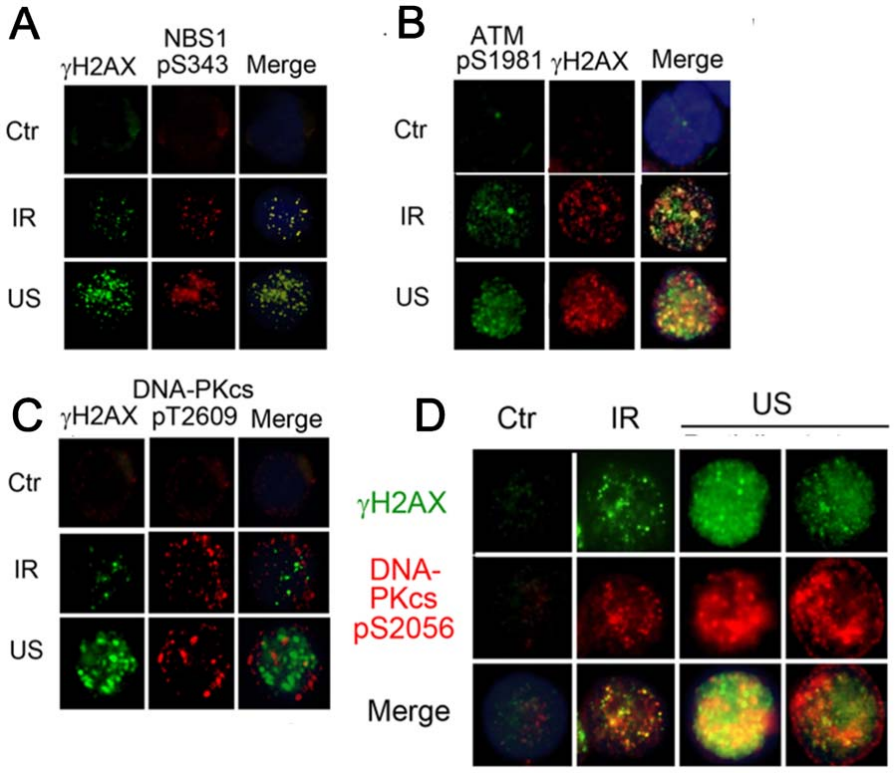
Differential ATM and DNA-PK signaling to γH2AX in response to US or IR. (A) Colocalization of distinct NBS1 pS343 foci to distinct γH2AX foci; (B) Colocalization of ATM pS1981 foci to γH2AX foci; (C) DNA-PKcs pT2609 foci largely independent of γH2AX foci. (D) US- and IR-induced DNA-PKcs pS2056 and γH2AX foci. US induced peri-nuclear high-fluorescent DNA-PKcs pS2056 foci (*right*) or were pan-nuclear with discrete foci *(left)*, whereas both foci after IR were distinct and colocalized. Fluorescent images were acquired 30 min after 0.3 W/cm^2^ US and 3 Gy IR in U937 cells.

To investigate, we undertook co-localization stains of γH2AX with two major kinases responsible for H2AX phosphorylation, ataxia-telangiectasia-mutated (ATM) and DNA-PKcs [22]. Figs. 2A–B show that the bulk of phospho-NBS1 and -ATM foci colocalized to γH2AX foci after both US *and* IR exposures, suggesting a general and coordinated recruitment of NBS1 and ATM to stress-induced DSBs [23], [24]. The pan-nuclear staining of γH2AX that occurs only after US exposure may arise through global ATM activation, perhaps via chromatin remodeling [25] in response to the nature of the US stress.

Furthermore, staining investigations of the two major phosphorylation clusters (T2609 and S2056) available to DNA-PKcs (for end-processing of DSB via non-homologous end joining (NHEJ) [26]–[29]) revealed (Figure 2C), that DNA-PKcs-pT2609 foci were largely independent of γH2AX after US and IR exposures, supporting earlier suggestions that NHEJ occurs separately from homologous recombination HR [30], [31]. Conversely, all IR-exposed cells displayed discrete, co-localized DNA-PKcs-pS2056/γH2AX+ nuclear foci (Figure 2D), also confirming previous reports that DNA-PKcs complements H2AX in response to IR [22]. Interestingly, observations on US induced γH2AX+ populations also exhibited overlapping regions of co-localization with DNA-PKcs, but additionally, a distinct signature of non-colocalized peri-nuclear DNA-PKcs-pS2056 (Figure 2D). Thus, preferential phosphorylation of DNA-PKcs-pS2056 may mediate both NHEJ repair in bulk-nuclear US-induced DSBs, but also signal to γH2AX presence around the nuclear periphery (see also Figure S6). The mechanism by which DNA-PKcs S2056 is distributed around the periphery of the nucleus remains unclear, however, this localization patterns of the DNA-PKcs S2056 may be one of the characteristic cellular responses to US-induced DSBs.

**Figure 3:**
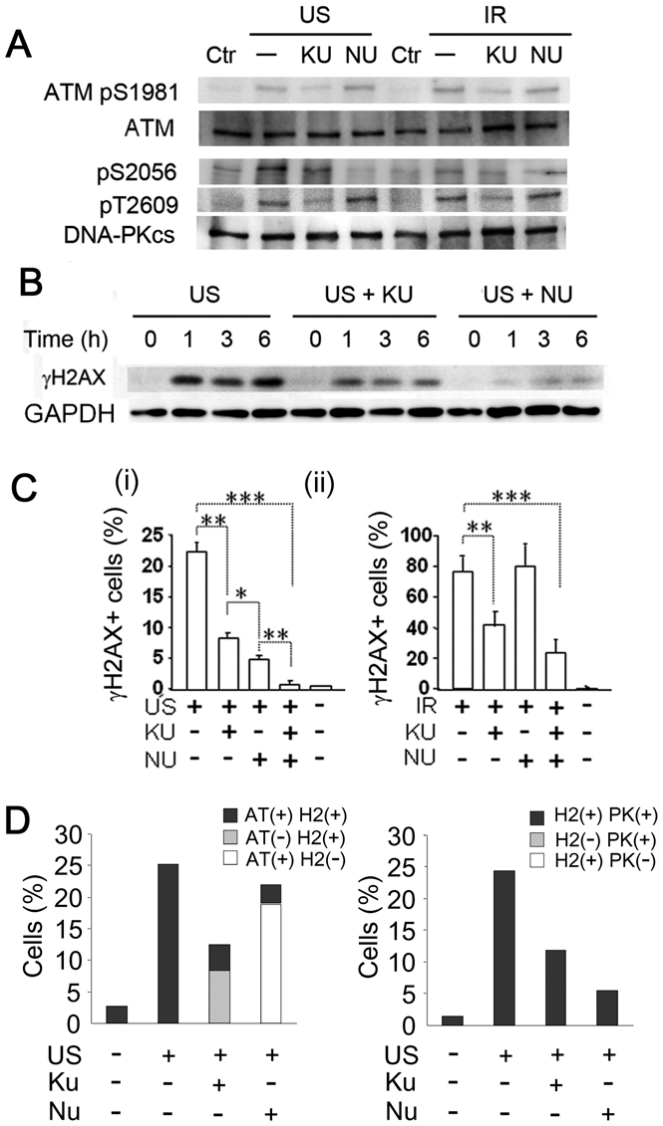
Effects of KU55933 (KU) and NU7026 (NU) on US- or IR-induced γH2AX signaling. (A) Immunoblots of U937 cell extracts 30 min after US or IR and effects of KU and NU on ATM pS1981 and DNA-PKcs pS2056/pT2609. (B) Greater suppression of US-induced γH2AX by NU than KU up to 6 h after US in U937 cells in WB analysis. (C) Effects of KU and/or NU: a greater suppression of US-induced γH2AX+ cells by NU than KU, and abrogation by KU-plus-NU in FCM analysis. (*n* = 3, mean ± s.d.). *, *P*<0.05; **, *P*<0.01; ***, *P*<0.001. Cells were treated with KU and/or NU (10 µmol/L) for 1 h before and after exposure to 0.3 W/cm^2^ US (i) or 10 Gy IR (ii). (D) DNA-PK preceded ATM for γH2AX induction by US: a preferential role of DNA-PK in γH2AX induction was determined by immunostaining. Cells were treated as Fig. 4C. ATM pS1981 (AT), DNA-PKcs pS2056 (PK), and γH2AX (H2) positive/negative cells were counted at least 100 cells in each experiment. The data shows the averages from 2 independent experiments.

We further evaluated the biochemical roles of ATM and DNA-PK by applying the respective pharmacological kinase inhibitors KU55993 (KU) and NU7026 (NU). Immuno-blots revealed that IR elicited a greater ATM phosphorylation than did US (Figure 3A), somewhat reflecting our earlier NCTA observation (Figure 1A). Notably however, we found that KU, but not NU, selectively reduced the phospho-ATM levels after US and IR (Figure 3A). Here, phosphorylation of DNA-PKcs-S2056 (pS2056) was greater after US than IR. As anticipated, NU inhibited S2056 phosphorylation significantly, whilst KU reduced ATM-dependent T2609 phosphorylation [26], after US and IR. Moreover, KU partially reduced US-induced DNA-PKcs-pS2056 in both immuno-blotting and immuno-staining (Figure 3B, D), suggesting crosstalk between ATM and DNA-PKcs-S2056 in response to US exposure.

Given that US appears to activate DNA-PK in preference to ATM, it is perhaps not surprising that NU was more effective in reducing US-induced γH2AX protein levels than was KU (as illustrated for the case of U937 cells (Figure 3B), and in the other cell lines tested (Figure S7)) –an observation that was further bolstered by complementary flow cytometry measurements (Figure 3C and Figure S8), which also demonstrated complete inhibition when using a KU/NU combination (Figure 3C(i)). Such pharmacological inhibitions were also confirmed by immuno-staining and reproduced in all cells (Figue 3D and Figures S7, S9). The dependence of DNA-PKcs on US-induced H2AX phosphorylation was also confirmed by comparing the US-response of DNA-PKcs defective glioblastoma cell lines (M059J) with that of its parental cell lines (M059K) (Figure S10). In summary, these findings strongly support a preferential role for DNA-PKcs over ATM, possibly without involvement of ATR, in the early signaling from US-induced DSBs to γH2AX, but with the directly opposite sense of signaling from IR-induced DSBs (Figure 3A, C) as has been shown previously [22].

**Figure 4:**
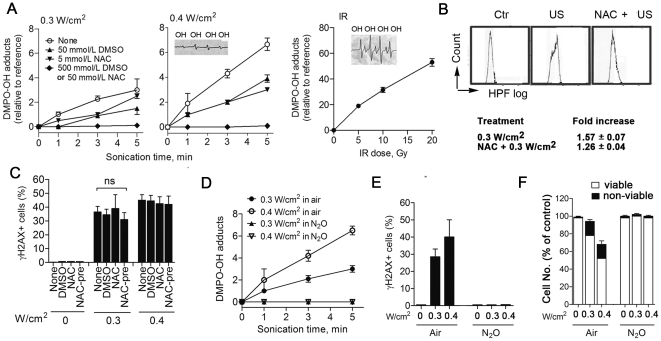
Linking mechanical US effect withinduction of γH2AX+ cells. (A) EPR detection of US- or IR-induced extra- and intracellular levels of OH• as DMPO-OH adducts (see Methods); Increase of OH• levels in DMPO solution (10 mmol/L) as a function of insonation time at 0.3 (*left*) and 0.4 (*middle*) W/cm^2^ US, or at IR dose of 5–20 Gy (*right, closed circle*). Induction of DMPO-OH adducts by 0.3 or 0.4 W/cm^2^ were reduced partially by 50 mmol/L DMSO (*upward triangle*) or 5 mmol/L NAC (*downward triangle*), and nullified by a 10-fold higher concentrations: 50 mmol/L NAC or 500 mmol/L DMSO (*closed diamond* for both). The insets show amplitudes of EPR signals of DMPO-OH. (B) FCM-based HPF assay for intracellular OH• levels immediately after 0.3 W/cm^2^ US in U937 cells. The histogram shift toward high-HPF fluorescence by OH• oxidation was small, and thus, an increase in mean fluorescence intensity (MFI) was 1.57±0.07 fold the control (*n* = 3, mean ± s.d.), with partial protection by pretreatment with 5 mmol/L NAC for 3 h before sonication. (C) No protective effects of 5 mmol/L DMSO or 5 mmol/L NAC (scavengers) added to cultures immediately before 0.3 and 0.4 W/cm^2^ US, or other 3 h-pretreatment with 5 mmol/L NAC (NAC-pre) against the induction of γH2AX+ U937 cells 30 min post-US. (*n* = 3, mean ± s.d. *ns* means *not significant*). (D–F) Saturated N_2_O gas caused the abrogation of US-induced events as follows: (D) The US exposure-time dependent induction of OH• in 10 mmol/L DMPO solution at 0.3 and 0.4 W/cm^2^ (*n* = 3, mean ± s.d.). : (E) The induction of γH2AX+ U937 cells 30 min after 0.3 and 0.4 W/cm^2^ (*n* = 3, mean ± s.d.). : (F) 20% non-viable cells (trypan blue dye exclusion test) and 30% loss in cell counts relative to control U937 cells 6 h after 0.3 or 0.4 W/cm^2^ US (*n* = 3, mean ± s.d.).

Finally, we wished to explore the physico-chemical mechanism in US induced bio-effects by further testing the hypothesis that sonochemistry plays a dominant role. Here, we evaluated the relationship between US-induced OH• radicals and DSB induction. We found that US-induced OH• levels (DMPO-OH adducts) in the aerobic DMPO solution increased in an intensity-, and exposure time-, dependent manner (Figure 4A) where induction rates of 1 and 2 DMPO-OH adducts per 0.3 and 0.4 W/cm^2^/min, respectively, were an order of magnitude smaller than the 30 adducts per 10 Gy (Figure 4A) observed for the case of IR exposure. Thus, the extracellular OH• level post-US was less than 10% of that occurring post-IR, even though comparable doses were applied (in terms of their potential to generate DNA damage (*viz* Figure 1A). Furthermore, addition of the radical scavengers DMSO and NAC at respectively high or low concentrations to DMPO solution, either abolished, or partially reduced US-induced OH• levels (Figure 4A; see caption, and Methods for further details). Next, we determined the intracellular OH• levels immediately after US using a hydroxyphenyl fluorescein (HPF) assay [32]. Here, the mean fluorescence intensity (MFI) from a shifted flow cytometry histogram was 1.57±0.07 immediately after exposure to 0.3 W/cm^2^ (Figure 4B), Thus, low levels of both extra- and intra-cellular OH• arising in response to US cannot fully account for the US induction of DSBs. Moreover, none of the radical scavengers was effective at suppressing US-induced γH2AX (Figure 4C). On the contrary, N_2_O gas, which is known to suppress inertial cavitation of US [3], completely nullified the induction of DMPO-OH adducts, γH2AX+ cells, and cell death (Figure 4D–F). These observations taken in totality compel us to the conclusion that US-mediated mechanical stress, rather than any sonochemically generated radical activity, generates genomic DSBs.

Here, we demonstrate for the first time that the mechanical action of US using intermediate level intensities can induce DSBs, which are announced by the presence of neutral comet tail and γH2AX foci amongst a blanket pan-nuclear γH2AX with peri-nuclear DNA-PKcs S2056. In addition, the present US intensities of 0.3 and 0.4 W/cm^2^ , which gave rise to 0.132 and 0.144 MPa peak acoustic pressures, respectively [16], are beyond the diagnostic US range (<0.1 MPa) [1] and we confirmed that US at 0.1 W/cm^2^ (0.082 MPa) could not induce DSBs (Fig. 1E). These results emphasize the safety of diagnostic US, especially if the following three points are taken into consideration: (i) very short pulses (a couple of microseconds) used in diagnosis, (ii) standing waves are unlikely to occur in *in vivo* exposures, and (iii) the attenuation of acoustic waves in the human body. In conclusion, we hope that these new and compelling observations will provide not only a firm biophysical and biochemical basis for understanding the genotoxic potential of US, but also guide future translation in terms of safety thresholds.

## Materials and Methods

### Chemicals and Cells

The DNA-PK inhibitor NU7026 and ATM inhibitor KU55933 were purchased from Calbiochem (Cambridge, UK). Human leukemia cell lines U937, Molt-4, and Jurkat-T were commercially obtained (Japanese Collection of Research Bioresources (JCRB) Cell Bank [32]. HL-60 was also obtained from JCRB Cell Bank (IFO50022). Cells were cultured in RPMI 1640 supplemented with 10% fetal bovine serum. Recombinant TRAIL/Apo2L was from PeproTech (London, UK); a pan-caspase inhibitor zVal-Ala-DL-Asp-fluoromethyl ketone (zVAD-fmk) was from Peptide Institute (Osaka, Japan); *N*-acetyl-L-cysteine (NAC) and propidium iodide (PI) were from Wako Pure Chemical (Tokyo, Japan); 4′,6-diamino-2-phenylindole (DAPI) and 5,5-demethyl-1-pyrroline-*N*-oxide (DMPO) were from Dojindo (Kumamoto, Japan).

### Sonication and Irradiation

Low-intensity-pulsed US with 100 Hz fixed pulse repetition frequency and 10% duty factor (thereafter designated as US) was generated using a 1.0 MHz acoustic setup [16], [33]. In insonation experiments, a 2 mL-aliquot at a fixed density of 1×10^6^ cells/mL in a 35-mm polyethylene culture dish (Corning, NY) was sonicated at 0.1–0.4 W/cm^2^ (devise-indicated intensities) for 1 min. These four intensities corresponded to 0.061, 0.105, 0.132 and 0.144 MPa peak acoustic pressures, respectively [16]. A rise of medium temperature during insonation was below 1°C [16]. For IR treatment, cells were irradiated with 3 Gy (to produce discrete IRIFs) or 10 Gy (a near-isoeffect dose for neutral comet tails induced by 0.3 and 0.4 W/cm^2^) at a dose rate of 5 Gy/min using a Model MBR-1520R-3 X-ray unit (Hitachi Medico Technology, Kashiwa, Japan).

### Neutral comet assay

Neutral comet tails (DSBs) were assessed in US-exposed cells using a Comet assay kit and electrophoresis unit (Trevigen) according to the manufacture's instruction. At least 50 cells per samples were analyzed by using a Comet Assay IV software (Leica Microsystems). The relative tail moment was given by the ratio of comet tail moments (mean ± SD) of treated cells to those of controls (ratio = 1.0).

### Immunodetection

For immunofluorescent images, paraformaldehyde-fixed control and treated cells were permeabilized/blocked with 2% BSA/0.05% Triton X-100/Tris-buffered saline, and immunostained for 2 h with primary monoclonal antibody (mAb): anti-phospho-H2AX S139 (γH2AX, Milipore), 1∶400 or anti-ATM pS1981, 1∶250 (Upstate Biotechnology) or primary polyclonal antibody (pAb), anti-phospho-H2AX S139 (γH2AX, Active Motif), 1∶500, anti-NBS1 pS343, 1∶1000 (Novus Biologicals), or anti-DNA-PKcs pT2609 or pS2056, 1∶250 or 1∶600 (Abcam), respectively. Then, cells were stained for 1.5 h with the secondary antibody: Alexa Fluor 488 anti-mouse F (ab′) IgG or Alexa Fluor 555 anti-rabbit F(ab′) IgG (Cell Signaling Technology), 1∶400. Finally, the nuclei were counterstained with 2 µg/mL DAPI, and the samples were mounted in Antifade™ (Molecular Probes). Fluorescent images were acquired using a BX-50 fluorescence microscopy (Olympus Optics).

For flow-cytometry (FCM), cells were fixed with 70% cold methanol overnight, then blocked with 2% BSA/0.05% Triton X-100/Tris-buffered saline and reacted with γH2AX mAb/Alexa Fluor 488 anti-mouse IgG (1∶400) to stain γH2AX+ cells, followed by incubation with 1 mg/mL RNase A and 50 µg/mL PI for allocating γH2AX+ cells to each of the PI-based cell-cycle phases. The samples were finally run on an Epics XL flow cytometer (Beckman Coulter).

For immunoblot analysis, whole-cell extracts were prepared in RIPA lysis buffer containing sodium orthovanadate and cocktail of protease inhibitors (Nacalai Tesque). High molecular weight molecules of ATM and DNA-PKcs were separated in 5% precast SDS-PAGE gels, whereas other lower molecular weight proteins were separated in 15% precast SDS-PAGE gels. After transfer, proteins on the Immobilon-P membranes (Millipore) were western-blotted by using the primary antibodies: γH2AX mAb, ATM pAb (Santacruz), ATM pS1981 mAb (Epitomics), DNA-PKcs pAb (Epitomics), DNA-PKcs pS2056 pAb, DNA-PKcs pT2609 mAb, caspase 3 pAb (Cell signaling) or GAPDH mAb (loading reference, Organon Teknika), and the secondary HRP-conjugated anti-mouse or anti-rabbit IgGs (Cell Signaling). Protein expression levels were visualized by an enhanced chemiluminescence (ECL) detection system (Nacalai Tesque), and images were acquired by a LAS-4000 luminescent image analyzer (Fuji Film).

### Detection of extra- and intra-cellular ROS

The levels of US- or IR-induced OH• in extracellular fluids were quantified by the electron paramagnetic resonance (EPR) spin-trapping method [3], [16]. For these, 2-mL aliquots of 10 mM DMPO were dispensed into 35-mm dishes and exposed to 0.3 and 0.4 W/cm^2^ for 1 to 5 min or to graded IR doses (5–20 Gy), followed by the immediate detection of DMPO-OH adduct signals using a RFR-30 EPR spectrometer (Radical Research). DMPO-OH adducts (OH•) were expressed as relative amounts to an internal reference (Mn^2+^). To detect intracellular OH•, cell-permeable hydroxyphenyl fluorescein (HPF) (Sekisui Medical) was used, which detects mainly OH• and marginally ONOO^—^ (∼1/10 the OH• amount) [31]. Cells were loaded with 5 nM HPF for 15 min at 37°C, and exposed to 0.3 W/cm^2^, followed by the immediate FCM analysis of US-induced intracellular OH•. Mean fluorescence intensity (MFI) of the oxidized probe was quantified to assess its fold increase over the control.

### Statistics

Data were presented as means ± s.d. Statistical significance between any two data sets was analyzed using unpaired Student's *t*-test with Microsoft Excel 2007.

## Supporting Information

Figure S1 **Assay for neutral comet tails in Jurkat, Molt-4 and HL-60 cells immediately after 0.4 W/cm^2^ US revealed the uneven broader distribution of 25–30, 35–50% and 10–23% cells to ranges of >3, 1.1–3, and 1 relative tail moments, respectively, compared to a rather uniform distribution of 80–90% majority cells to a smaller range of 1.1–3 relative moments after 10 Gy IR.** Relative tail moment of 1.0 represents no induced DSBs as in the control cells. After 0.3 W/cm^2^, similarly, 10–25, 30–40% and ∼50% cells incurred >3, 1.1–3 and 1 (no DSBs) relative tail moments, respectively. These results recapitulate the findings in U937 cells (Fig. 1A, C).
(PDF)

Figure S2 **Fluorescence images showed pan-nuclear γH2AX pattern 30 min after 0.4 W/cm^2^ US, but no γH2AX+ cells after 0.1 W/cm^2^ in U937 cells.** Cells with 10 Gy of IR were used as positive control for γH2AX staining.
(PDF)

Figure S3 **Fluorescence images of γH2AX in U937, Jurkat, Molt-4 and HL-60 cells without US- or IR-exposure.** Quantified data are shown in Fig. 1E.
(PDF)

Figure S4 **Representative FCM histograms showed the induction and decline of γH2AX+ U937 cells with time up to 6 h after 0.3 or 0.4 W/cm^2^ (1 min) US or 10 Gy IR.** Time-course changes in γH2AX+ cells after US or IR (Fig. 1h) came from mean fluorescence of the histograms (shadowed). Note maximal γH2AX+ fractions at 0.5 or 1 h, followed by their decreases later, with some persistent γH2AX+ fractions around 6 h post-stress.
(PDF)

Figure S5 **Different H2AX responses to US and death-receptor ligand TRAIL in U937, Jurkat, Molt-4, and HL-60 cells.** (A) Time-dependent increases in γH2AX protein expression and p17/p19 active forms of cleaved caspase-3, an essential apoptotic marker, after addition of 0.1 mg/mL TRAIL. (B) zVAD-suppressive caspase-3 cleavage in U937, Jurkat, Motl-4 and HL-60 cells: inhibition of caspase-3 cleavage by treatment with zVAD-fmk for 6 h after 0.3 W/cm^2^ US (upper) or 3 h after TRAIL (bottom). Z-VAD FMK were pretreated 1 h before TRAIL treatement. (C) TRAIL-induced, apoptotic DSB-driven γH2AX+ cells but not DSB-driven γH2AX+ cells early 30 min after 0.3 W/cm^2^ US, were abrogated by treatment of all cell lines with 100 µmol/L zVAD-fmk. Blue DAPI color was changed to red for easy yellow visualization in the merge with green γH2AX image by using Adobe PHOTOSHOP Elements 2.0. (Adobe Systems).
(PDF)

Figure S6 **Immunofluorescence analyses of US- and IR-induced DNA-PKcs pS2056 and γH2AX foci.** Pan-nuclear green γH2AX foci and highly red-fluorescent DNA-PKcs pS2056 foci after US, but low-fluorescent distinct γH2AX and DNA-PKcs pS2056 foci after IR. Red arrows indicated cells with peri-nuclear DNA-PKcs pS2056 foci observed in sonicated cells but not in irradiated cells. Magnified images were in Fig. 2D.
(PDF)

Figure S7 **Western blot analyses showing effects of Ku55933 (KU) and/or Nu7026 (NU) on γH2AX 1 h after US in Jurkat, Molt-4, and HL-60 cells.** Cells were pretreated with 10 mmol/L of KU and/or NU 1 h before US.
(PDF)

Figure S8 **Typical FCM histograms showing US-induced γH2AX in the presence or absence of Ku55933 (KU) and/or Nu7026 (NU).** Distributions of cell-cycle phase were determined by staining with propidium iodide. Note that US-induced γH2AX were not restricted in S phase and suppressive effects of KU and/or NU on γH2AX were identified throughout cell-cycle phases.
(PDF)

Figure S9 **Typical images showing US-induced γH2AX, phospho-ATM at S1981, phospho-DNA-PKcs at S2056 in the presence or absence of Ku55933 (KU) and/or Nu7026 (NU).** The effect of KU or NU on expression of these proteins was quantified as in Fig. 4D.
(PDF)

Figure S10 **Typical FCM histograms showing US-induced γH2AX in DNA-PKcs proficient M059K cells but not in DNA-PKcs deficient M059J cells.** These adherent cell lines were resuspended by trypsinization and then sonicated at 0.4 W/cm^2^ for 60 sec in culture medium. Cells were collected in plastic tubes immediately after sonication then incubated for 30 min followed by fixation. Note that γH2AX induction by US-exposure was not restricted in leukemia cell lines and that DNA-PKcs was involved in H2AX phosphorylation in glioblastoma cell lines.
(PDF)
