# Supplementary figures and images for: DNA Double-Strand Breaks Induced by Cavitational Mechanical Effects of Ultrasound in Cancer Cell Lines
Source: PLoS One. 2012 Jan 3;7(1):e29012. doi: 10.1371/journal.pone.0029012 (PMC3250400; doi:10.1371/journal.pone.0029012)

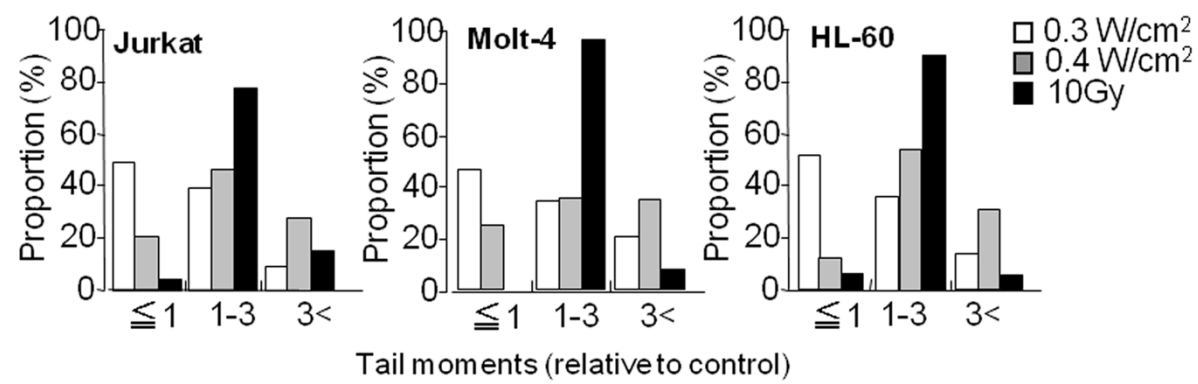

Supplementary Figure. 1

Supplement: Figure S1 — Assay for neutral comet tails in Jurkat, Molt-4 and HL-60 cells immediately after 0.4 W/cm2 US revealed the uneven broader distribution of 25–30, 35–50% and 10–23% cells to ranges of >3, 1.1–3, and 1 relative tail moments, respectively, compared to a rather uniform distribution of 80–90% majority cells to a smaller range of 1.1–3 relative moments after 10 Gy IR. Relative tail moment of 1.0 represents no induced DSBs as in the control cells. After 0.3 W/cm2, similarly, 10–25, 30–40% and ∼50% cells incurred >3, 1.1–3 and 1 (no DSBs) relative tail moments, respectively. These results recapitulate the findings in U937 cells (Fig. 1A, C). (PDF) [file pone.0029012.s001.pdf]

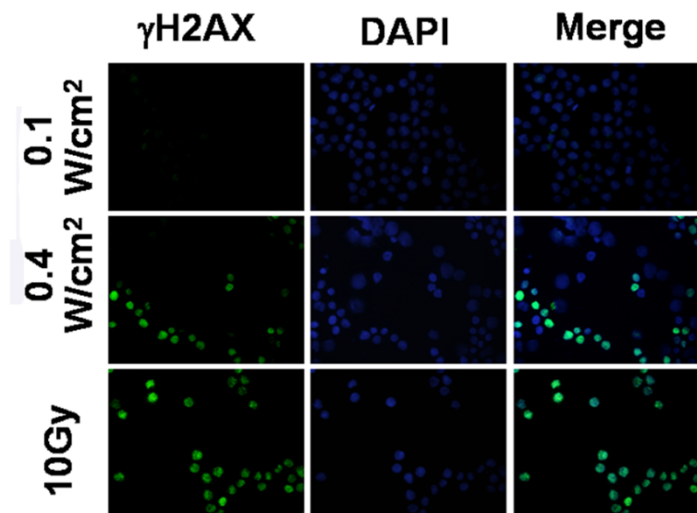

Supplementary Figure. 2

Supplement: Figure S2 — Fluorescence images showed pan-nuclear γH2AX pattern 30 min after 0.4 W/cm2 US, but no γH2AX+ cells after 0.1 W/cm2 in U937 cells. Cells with 10 Gy of IR were used as positive control for γH2AX staining. (PDF) [file pone.0029012.s002.pdf]

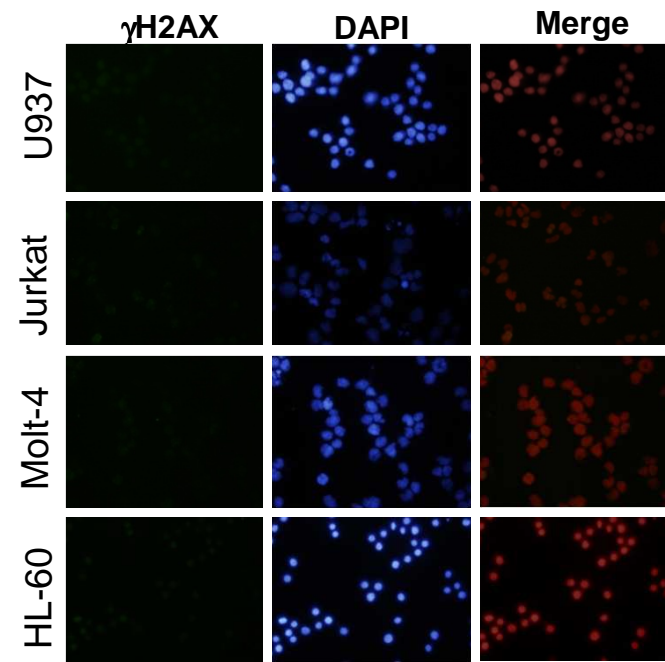

Supplementary Figure. 3

Supplement: Figure S3 — Fluorescence images of γH2AX in U937, Jurkat, Molt-4 and HL-60 cells without US- or IR-exposure. Quantified data are shown in Fig. 1E. (PDF) [file pone.0029012.s003.pdf]

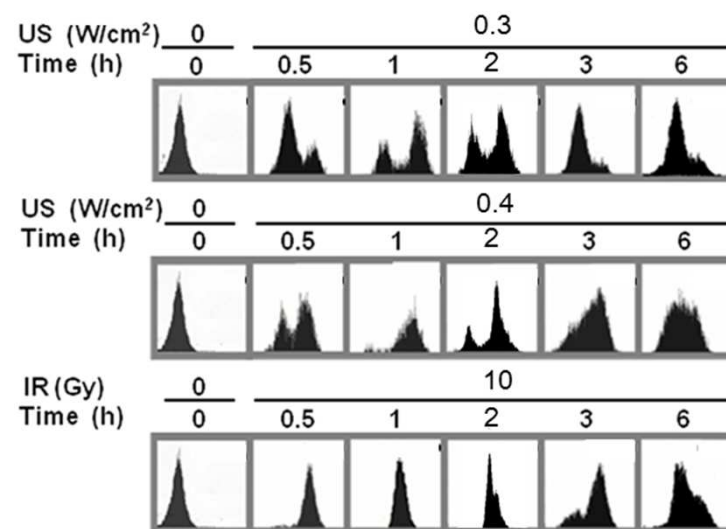

Supplementary Figure. 4

Supplement: Figure S4 — Representative FCM histograms showed the induction and decline of γH2AX+ U937 cells with time up to 6 h after 0.3 or 0.4 W/cm2 (1 min) US or 10 Gy IR. Time-course changes in γH2AX+ cells after US or IR (Fig. 1h) came from mean fluorescence of the histograms (shadowed). Note maximal γH2AX+ fractions at 0.5 or 1 h, followed by their decreases later, with some persistent γH2AX+ fractions around 6 h post-stress. (PDF) [file pone.0029012.s004.pdf]

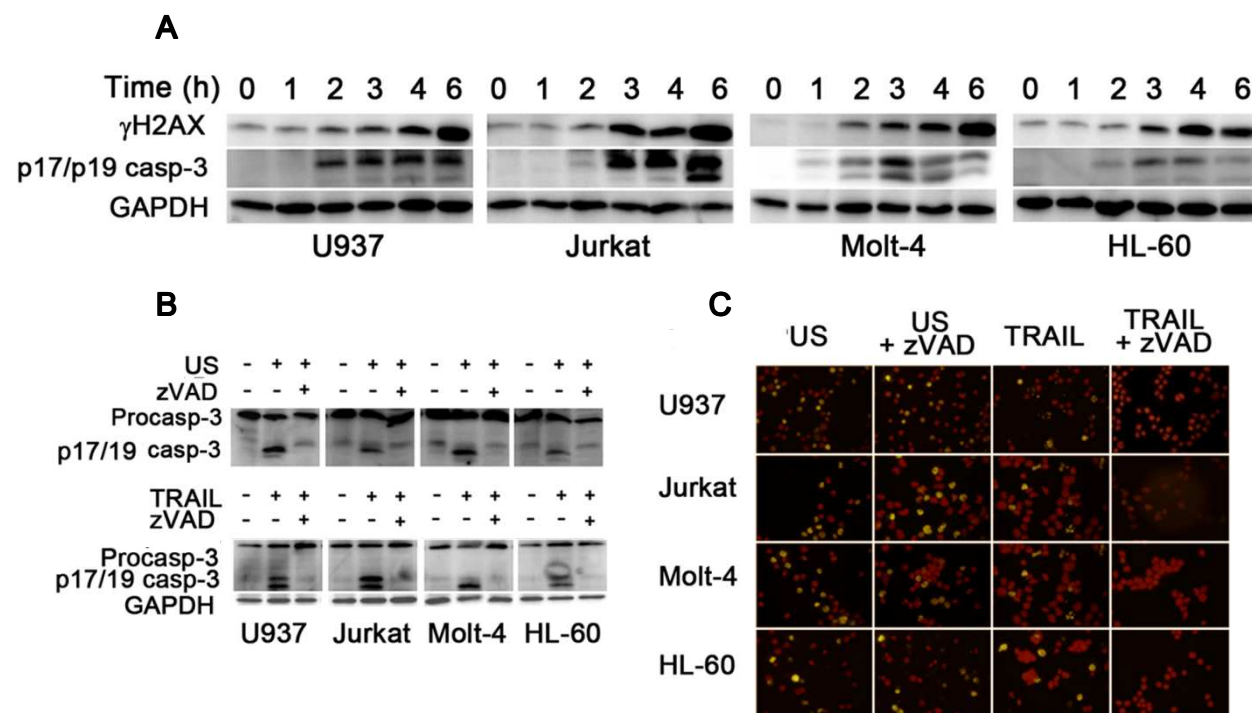

Supplementary Figure. 5

Supplement: Figure S5 — Different H2AX responses to US and death-receptor ligand TRAIL in U937, Jurkat, Molt-4, and HL-60 cells. (A) Time-dependent increases in γH2AX protein expression and p17/p19 active forms of cleaved caspase-3, an essential apoptotic marker, after addition of 0.1 mg/mL TRAIL. (B) zVAD-suppressive caspase-3 cleavage in U937, Jurkat, Motl-4 and HL-60 cells: inhibition of caspase-3 cleavage by treatment with zVAD-fmk for 6 h after 0.3 W/cm2 US (upper) or 3 h after TRAIL (bottom). Z-VAD FMK were pretreated 1 h before TRAIL treatement. (C) TRAIL-induced, apoptotic DSB-driven γH2AX+ cells but not DSB-driven γH2AX+ cells early 30 min after 0.3 W/cm2 US, were abrogated by treatment of all cell lines with 100 µmol/L zVAD-fmk. Blue DAPI color was changed to red for easy yellow visualization in the merge with green γH2AX image by using Adobe PHOTOSHOP Elements 2.0. (Adobe Systems). (PDF) [file pone.0029012.s005.pdf]

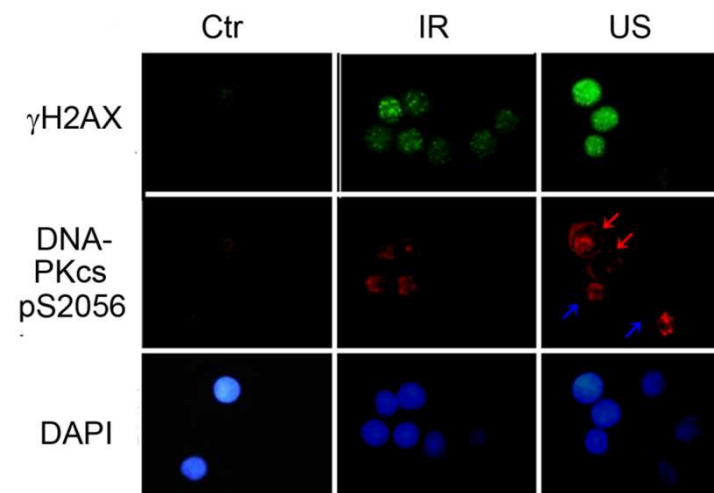

Supplementary Figure. 6

Supplement: Figure S6 — Immunofluorescence analyses of US- and IR-induced DNA-PKcs pS2056 and γH2AX foci. Pan-nuclear green γH2AX foci and highly red-fluorescent DNA-PKcs pS2056 foci after US, but low-fluorescent distinct γH2AX and DNA-PKcs pS2056 foci after IR. Red arrows indicated cells with peri-nuclear DNA-PKcs pS2056 foci observed in sonicated cells but not in irradiated cells. Magnified images were in Fig. 2D. (PDF) [file pone.0029012.s006.pdf]

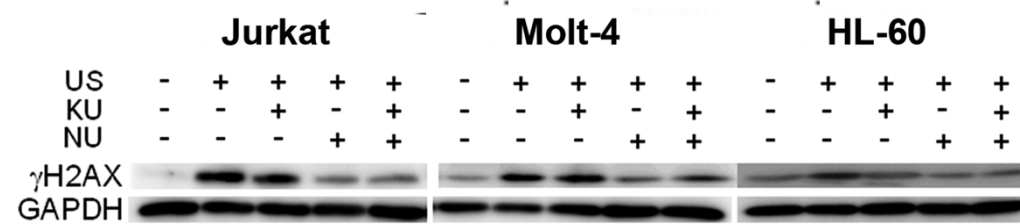

Supplementary Figure.7

Supplement: Figure S7 — Western blot analyses showing effects of Ku55933 (KU) and/or Nu7026 (NU) on γH2AX 1 h after US in Jurkat, Molt-4, and HL-60 cells. Cells were pretreated with 10 mmol/L of KU and/or NU 1 h before US. (PDF) [file pone.0029012.s007.pdf]

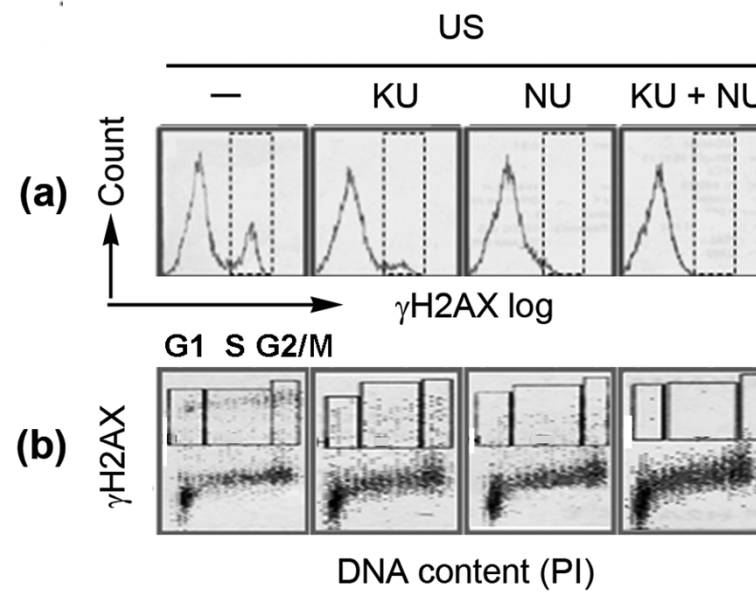

Supplementary Figure. 8

Supplement: Figure S8 — Typical FCM histograms showing US-induced γH2AX in the presence or absence of Ku55933 (KU) and/or Nu7026 (NU). Distributions of cell-cycle phase were determined by staining with propidium iodide. Note that US-induced γH2AX were not restricted in S phase and suppressive effects of KU and/or NU on γH2AX were identified throughout cell-cycle phases. (PDF) [file pone.0029012.s008.pdf]

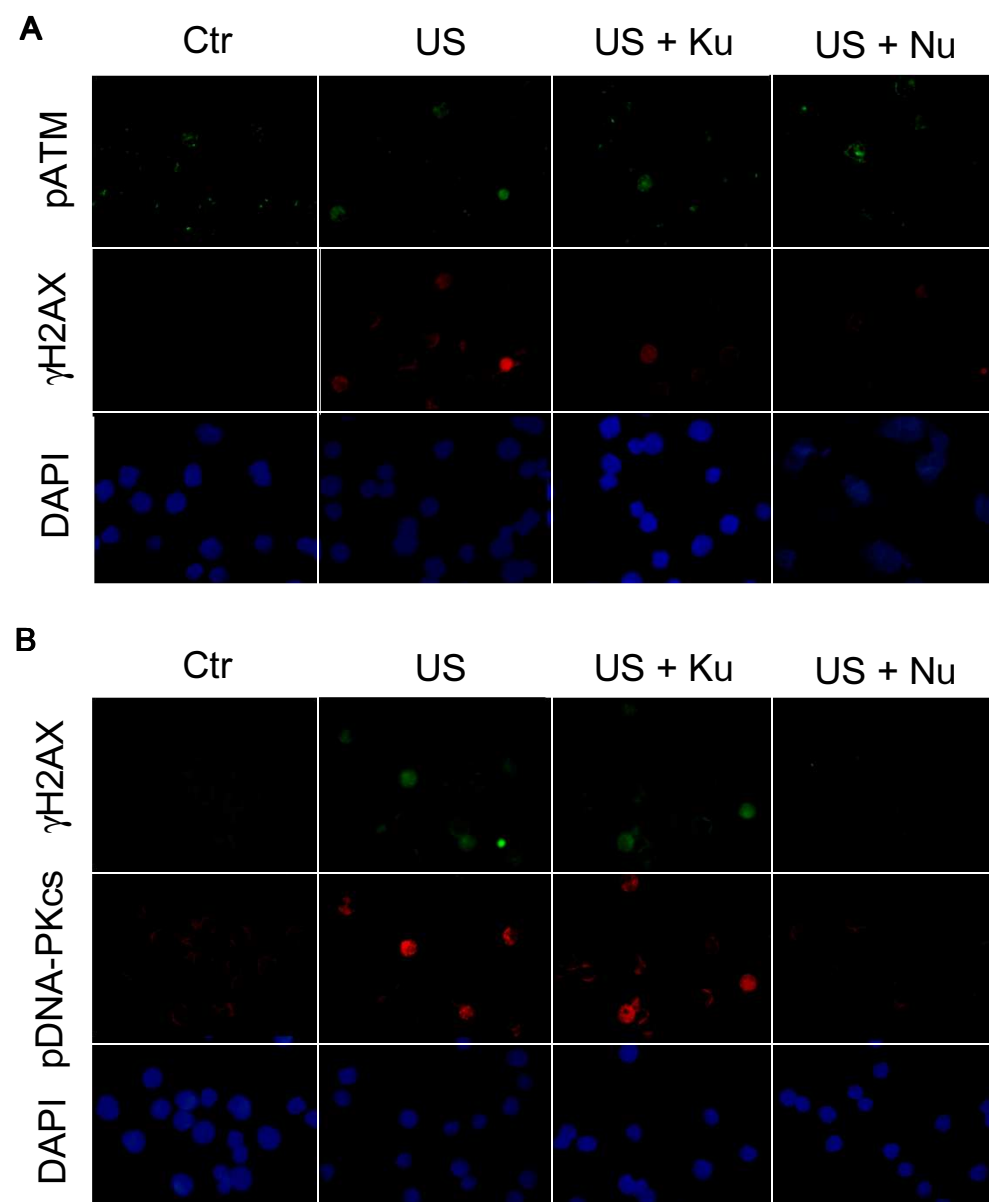

Supplementary Figure . 9

Supplement: Figure S9 — Typical images showing US-induced γH2AX, phospho-ATM at S1981, phospho-DNA-PKcs at S2056 in the presence or absence of Ku55933 (KU) and/or Nu7026 (NU). The effect of KU or NU on expression of these proteins was quantified as in Fig. 4D. (PDF) [file pone.0029012.s009.pdf]

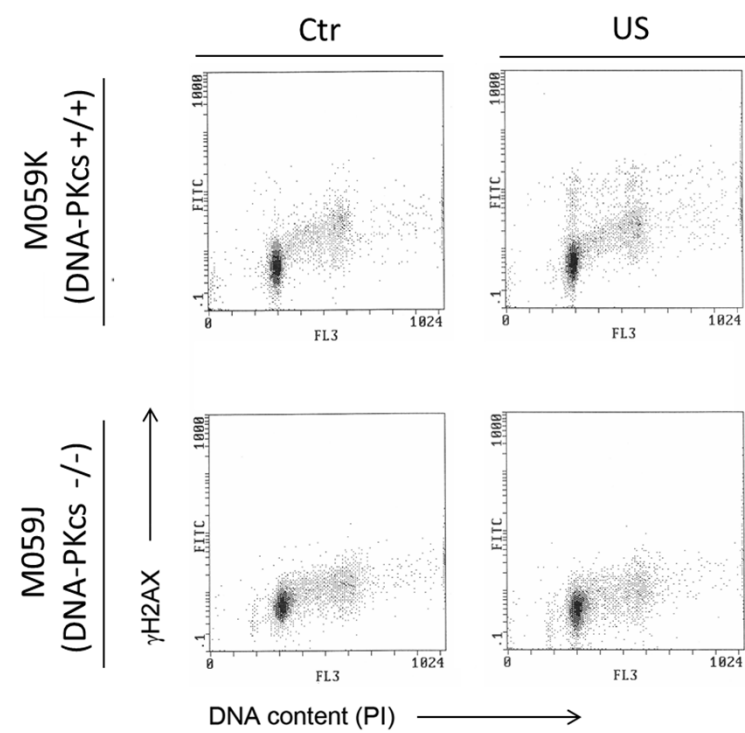

Supplementary Figure. 10

Supplement: Figure S10 — Typical FCM histograms showing US-induced γH2AX in DNA-PKcs proficient M059K cells but not in DNA-PKcs deficient M059J cells. These adherent cell lines were resuspended by trypsinization and then sonicated at 0.4 W/cm2 for 60 sec in culture medium. Cells were collected in plastic tubes immediately after sonication then incubated for 30 min followed by fixation. Note that γH2AX induction by US-exposure was not restricted in leukemia cell lines and that DNA-PKcs was involved in H2AX phosphorylation in glioblastoma cell lines. (PDF) [file pone.0029012.s010.pdf]
